# *Plasmodium falciparum* and soil-transmitted helminth co-infections among children in sub-Saharan Africa: a systematic review and meta-analysis

**DOI:** 10.1186/s13071-016-1594-2

**Published:** 2016-06-15

**Authors:** Abraham Degarege, Emir Veledar, Dawit Degarege, Berhanu Erko, Mathieu Nacher, Purnima Madhivanan

**Affiliations:** Department of Epidemiology, Robert Stemple College of Public Health, Florida International University, Miami, USA; Aklilu Lemma Institute of Pathobiology, Addis Ababa University, Addis Ababa, Ethiopia; Ethiopian Ministry of Health Office, Addis Ababa, Ethiopia; Université de Guyane, EA3593 Cayenne, French Guiana; Public Health Research Institute of India, Mysore, India

**Keywords:** Soil-transmitted helminths, *Plasmodium*, Co-infection, Children, Sub-Saharan Africa

## Abstract

**Background:**

The epidemiology of soil-transmitted helminth (STH) and *Plasmodium* co-infections need better understanding. The findings of the individual studies are inconclusive. A systematic review was conducted to synthesize evidence on the association of STH infection with the prevalence and density of *Plasmodium falciparum* infection, and its effect on anaemia among children in sub-Saharan Africa (SSA).

**Methods:**

Relevant studies published before March 6, 2015 were identified by searching Medline (via Pubmed), Embase, Cochrane Library and CINAHL without any language restriction. Studies on *P. falciparum* and STH co-infection among children in SSA except for case studies were included in this study. Studies were screened for eligibility and data extracted independently by two authors. The primary outcome assessed was the prevalence of *P. falciparum* infection and the secondary outcomes included *P. falciparum* density and prevalence of anaemia. Heterogeneity was assessed using Cochrane Q and Moran’s *I*^2^ and publication bias was evaluated using Egger test. A random-effects model was used to estimate the summary odds ratio (OR) and the corresponding 95 % confidence intervals (CI).

**Results:**

Out of 2985 articles screened, 11 articles were included in the systematic review; of these seven were considered in the meta-analysis. Of the 11 studies with 7458 study participants, seven were cross-sectional, one prospective cohort and three were randomized controlled trials. Four studies examined the outcome for hookworms, one for *Ascaris lumbricoides* and six for pooled (at least one) STH species. Eight studies measured prevalence/incidence of uncomplicated *P. falciparum* infection, two calculated prevalence of asymptomatic *P. falciparum* infection, three evaluated *P. falciparum* density and four considered prevalence of *P. falciparum* infection related anaemia/mean haemoglobin reduction. The odds of asymptomatic/uncomplicated *P. falciparum* infection were higher among children infected with STH than those uninfected with intestinal helminths (summary Odds Ratio [OR]: 1.4; 95 % Confidence Interval [CI]: 1.05–1.87; *I*^2^ = 36.8 %). *Plasmodium falciparum* density tended to be higher among children infected with STH than those uninfected with intestinal helminths. However, STH infection was associated with lower odds of *P. falciparum* infection related anaemia (summary OR: 0.5; 95 % CI: 0.21–0.78; *I*^2^ = 43.3 %).

**Conclusions:**

The findings suggest that STH infection may increase susceptibility to asymptomatic/uncomplicated *P. falciparum* infection but may protect malaria-related anaemia in children. Future studies should investigate the effect of STH infection upon the incidence of severe *P. falciparum* infection among children in SSA.

**Electronic supplementary material:**

The online version of this article (doi:10.1186/s13071-016-1594-2) contains supplementary material, which is available to authorized users.

## Background

In 2015, about 214 million people globally were infected with *Plasmodium* spp. responsible for malaria and 438,000 died from it [1]. Close to 88 % of these cases and 90 % of the deaths were in sub-Saharan Africa (SSA) [1]. Most malaria cases and deaths due the disease in SSA are attributable to *P. falciparum* infection [1]. In addition, about two billion people are infected with soil-transmitted helminths (STH) worldwide with the greatest numbers occurring among children in SSA [2, 3]. Approximately 807–1121 million of these STH infections are with *Ascaris lumbricoides*, 604–795 million with *Trichuris trichiura* and 576–740 million with hookworms [3]. STH and *Plasmodium* spp. infections usually overlap in distribution and share similar ecological transmission risks leading to populations being at increased risk of co-infection with both parasite groups [4]. The prevalence of *Plasmodium* and STH co-infection is particularly high among children in SSA [4]. For example, more than 25 % of school-aged children in SSA, were estimated to be at risk of *P. falciparum* and hookworm co-infection [5].

STH and *Plasmodium* parasites activate different modes of the immune system in the human body [6]. It is assumed that helminth infections, can downregulate immune responses to *Plasmodium* pathogens [6]. Helminths may also interact with *Plasmodium* spp. through other mechanisms including resource competition and direct interference [7]. Hence, occurrence of STH infection could increase susceptibility to *Plasmodium* infection spp. and related clinical outcomes [6]. However, findings on whether and how STH and *Plasmodium* spp. interact within humans when there are co-infections, are heterogeneous [8–11]. Some studies reported protective effect of STH infection upon subsequent *Plasmodium* infection [8, 9], while others document increased *Plasmodium* infection in children infected with STH [10, 11]. *Plasmodium* spp. and helminths affect haemoglobin levels in different ways and exert an additive effect when they co-exist, leading to an increased risk of iron-deficiency anaemia among co-infected individuals [4]. However, studies examining this relationship have shown conflicting results [11–13].

In addition, the reason why malaria vaccine trials often failed to induce effective protection in areas where STH infection is common, also remains to be answered [14, 15]. Downregulation of Th1 immune response during STH infection may impede the development of vaccine-induced protective immunity against malaria [6]. Therefore, in SSA regions where there are high rates of *Plasmodium* spp. and STH co-infections, the incidence and the clinical course of malaria could be affected in children. Despite such uncertainty, children living in most areas of the SSA region are regularly treated for STH infection with anthelminthic drugs to reduce morbidity due to infection with these parasites [16].

In the two previous narrative reviews [17, 18], the authors assessed the nature of interaction between helminth and *Plasmodium* spp. infection. However, studies included in these reviews were highly heterogeneous regarding the types of helminth species, study participants and geographical locations of the study areas, which could potentially have affected the conclusions. Therefore, a systematic review was conducted to assess the impact of STH on asymptomatic/uncomplicated *P. falciparum* infection, parasite density and related anaemia among children in SSA. The information will help understand the effect of *P. falciparum* and STH co-infection on the epidemiology of malaria among children living in SSA. This will contribute to planning integrated disease control strategies and reveal the implications of mass deworming programs in the course of malarial disease in SSA.

## Methods

This systematic review and meta-analysis were planned, conducted and reported in accordance to the PRISMA guidelines [19] (see Additional file 1).

### Eligibility criteria

All study designs except case studies were included irrespective of language. To be included in the review, studies should have been among children living in SSA; reported prevalence or incidence of malaria, prevalence of malaria-related anaemia or *Plasmodium* density stratified by the presence of at least one STH species including *Ascaris lumbricoides*, hookworms or *Trichuris trichuria* and absence of intestinal helminth infection. Studies that reported immunology of malaria and STH co-infection were also included if they contained information about malaria prevalence/incidence, *Plasmodium* density and malaria-related anaemia stratified by presence of at least one STH species and absence of intestinal helminth infection. Other studies from SSA in the general population that reported data on children separately were also considered for inclusion. Conference abstracts, gray literature and unpublished studies were excluded. In addition, studies were excluded from meta-analysis when sufficient data on malaria prevalence/incidence, *Plasmodium* density and malaria-related anaemia were not available for children who were infected with STH and uninfected with intestinal helminths.

### Search methods for identification of studies

Eligible studies were identified by searching Pubmed, Embase, Cochrane Library, CINAHL databases through March 6, 2015. The keywords “malaria” OR “*Plasmodium*” OR “*Plasmodium falciparum*” OR “*Plasmodium vivax*” in combination with helminth “soil-transmitted helminth” OR “geohelminth” OR “*Ascaris*” OR “*Ascaris lumbricoides*” OR “*Trichuris*” OR “*Trichuris trichiura*” OR “hookworm” OR “*Ancylostoma*” OR “*Ancylostoma duodenale*” OR “*Necator*” OR “*Necator americanus*” were used (see Additional file 2: Table S1 for details). Literature search was limited to humans. Furthermore, we searched the African Journals Online database and the reference lists of the previous reviews on malaria and helminth co-infection [4, 6, 17, 18, 20]. Two reviewers (AD and DD) screened articles for eligibility after excluding the duplicates. First screen of titles/abstracts was based on inclusion/exclusion criteria followed by a second screen of full text review of select articles.

### Types of outcome measures

The primary outcome was prevalence of asymptomatic/uncomplicated *P. falciparum* infection. Uncomplicated (mild) or asymptomatic (no symptoms) *P. falciparum* infection was defined as detection of *P. falciparum* in the blood smear examined by microscopy without manifestation of any severe malaria symptoms [21].

The secondary outcomes measured were: (i) *P. falciparum* density defined as number of parasites counted per microliter of blood; and (ii) *P. falciparum* infection related anaemia defined as haemoglobin level below the cut-off values set by WHO: 11.0 g/dl for children 6–59 month-old; 11.5 g/dl for children 5–11 year-old; 12.0 g/dl for children 12–14 year-old [22].

### Data collection

Relevant information from the selected articles was extracted and entered into a standardized excel sheet by two authors (AD and DD) independently. Any differences were resolved on further discussion with the third author (EV) before including the data in the review. The following data were collected from each included study: author, year of publication, study area, study design, sample size, STH and *Plasmodium* species investigated, outcome measured, prevalence of STH and *Plasmodium* spp. co-infection, prevalence of malaria-related anaemia, and *Plasmodium* spp. density in children infected with STH compared to those who were not infected with intestinal helminths.

### Statistical analysis

Odds ratio was used as a measure of association. Information about adjusted odds ratio (aOR) of asymptomatic/uncomplicated *P. falciparum* infection and related anaemia along with 95 % confidence interval (CI) were collected for children who were infected with STH and uninfected with intestinal helminths. When adjusted measures were not reported, raw data for only malaria-positive, only STH-positive, malaria- and STH-positive, and intestinal helminth- and malaria-negative were extracted from the tables, figures, texts or summary data of the articles. This information was used to estimate the crude odds of asymptomatic/uncomplicated *P. falciparum* infection and related anaemia in children infected with STH compared to those uninfected with intestinal helminths along with their 95 % confidence intervals. The log OR and the standard error (SE) of the log OR were estimated using generic inverse variance weighting method [23] and then the summary estimate (summary-odds ratio) was estimated. Heterogeneity was tested using Cochrane Q (Chi-square) and Moran’s *I*^*2*^ (Inconsistency). Publication bias was evaluated using Egger’s regression test (bias if *P* < 0.1) [24]. Random-effects model was used to estimate the summary Mantel-Haenszel odds ratio of asymptomatic/uncomplicated *P. falciparum* infection in children infected with STH compared to those without intestinal helminth infection. All statistical analyses were conducted using packages in R (version 2.15, R Foundation for Statistical Computing, Vienna, Austria) [25].

### Quality and bias assessment

The methodological quality and the risk of bias of the studies included in the review were judged using the quality assessment tool for quantitative studies by the effective public health practice project, which determines study quality on the basis of selection of the study participants, study design, confounder, blinding, data collection methods and withdrawals and drop-outs comparability [26].

## Results

### Search results

**Fig. 1:**
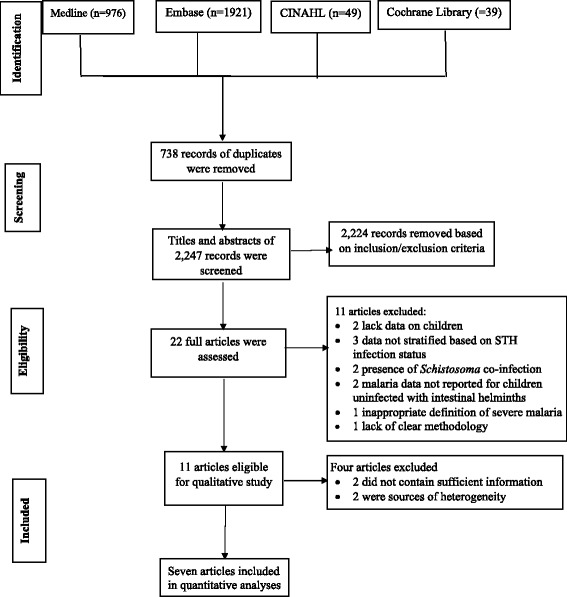
Flowchart showing the selection and exclusion of the studies

A flowchart of the identification of relevant studies is shown in Fig. 1. A total of 2985 articles were obtained from searching four databases, 738 were duplicates and 2225 articles that did not meet the selection criteria were excluded. The remaining 22 articles were obtained for full-text review [10, 11, 27–46]. Of these, eleven articles contained insufficient information and were excluded from the review [10, 11, 38–46]. The remaining eleven studies were considered for the qualitative analysis in this review [27–37]; of these, seven were included in the meta-analysis [28, 29, 31–33, 36, 37].

### Study characteristics

Characteristics of the included studies are presented in Table 1. The 11 studies with 7458 participants included in the review were conducted in Cameron, Côte d’Ivoire, Kenya, Nigeria, Tanzania, Senegal and Zanzibar (Table 1). Out of the 11 studies, seven were cross-sectional studies [27–30, 32, 35, 36], one prospective cohort study [33] and three randomized controlled trials [31, 34, 37]. The studies also differed in STH species that had been examined: four studies reported information on hookworms [27–30], one study on *A. lumbricoides* [31] and six studies reported on pooled (at least one) STH species [32–37]. In addition, the 11 studies differed in the type of outcomes assessed: eight studies measured the prevalence/incidence of uncomplicated *P. falciparum* infection [27, 29, 31, 33–37], two reported prevalence of asymptomatic *P. falciparum* infection [28, 32], three evaluated *Plasmodium* density [30, 36, 37] and four considered prevalence of *P. falciparum* infection related anaemia/mean haemoglobin reduction [29, 32, 35, 37]. Except for one study [34], all studies controlled age and sex while examining the relationship between *P. falciparum* and STH infection. Some studies additionally controlled the effect of socioeconomic status [29, 31, 37], area of residence or village [29, 32, 34, 37], presence of other infections [27, 37], nutritional status [29], insect side treated net use [34] and household condition [33].

**Table 1:** Characteristics of the included studies

Reference	Study area	Sample size (Age range in years)	Study design	STH species	*Plasmodium* spp. diagnosed	Outcomes measured/reported	Prevalence of co-infection (%)	Magnitude of outcomes (infected with STH *vs* uninfected with intestinal helminths)	Variables used for adjusting
[31]	Nigeria	690 (1/2–6)	RCT	*Al*	*Pf*	1. *Pf* prevalence 2. Anaemia prevalence 3. 3. *Plasmodium* density	42.9	1. Similar (OR: 1.30; 95 % CI: 0.91–1.86) 2. Lower (OR: 0.50; 95 % CI: 0.28–0.87) 3. Similar (*P* = 0.965)	Age, sex and SES
[34]	Kenya	387 (1–6)	RCT	*Al* + *Hw* + *Tt*	*Pf*	*Pf* incidence	na	Similar (RR: 0.83; 95 % CI: 0.51–1.33)	Age, village and ITN use
[27]	Tanzania	1,546 (3–13)	CS	*Hw*	*Pf*	*Pf* prevalence	2.3	Similar (OR: 1.19; 95 % CI: 0.86–1.63)	Age, sex, schools and other infections
[35]	Zanzibar	2322 (1/2–2)	CS	*Al* + *Hw* + *Tt*	~80 % *Pf*	*Pf* prevalence	5.1	Lower (OR: 0.63; 95 % CI: 0.50–0.80)	Age, sex and fever
[28]	Tanzania	400 (8–16)	CS	*Hw*	*Pf*	*Pf* prevalence	2.3	Similar (AOR: 1.35; 95 % CI: 0.49–3.72)	Age and sex
[36]	Cameroon	425 (0–14)	CS	*Al* + *Hw* + *Tt*	*Pf*	1. *Pf* prevalence 2. *Plasmodium* density 3. Anaemia prevalence	24.7	1. Similar (OR: 1.00; 95 % CI: 0.65–1.53) 2. Higher (*P* < 0.001) 3. Similar (OR: 1.19; 95 % CI: 0.642.21)	Age and sex
[29]	Côte d’Ivoire	324 (1/2–2; 6–8; 15–25)	CS	*Hw*	*Pf*	1. *Pf* prevalence 2. Anaemia prevalence	27.9	1. Higher (in 6–8 year-old) (AOR: 7.47–95 % CI: 1.84–30.32) 2. Lower (OR: 0.23; 95 % CI: 0.06–0.83)	Age, sex, SES, nutrition status, inflammation status and area of residence
[32]	Cameroon	263 (4–12)	CS	*Al* + *Tt*	*Pf*	1. *Pf* prevalence 2. Haemoglobin levels	13.9	1. Similar (OR: 1.29; 0.60–2.76) 2. Similar	Age, sex and area of residence
[30]	Tanzania	578 (7.96 ± 1.4)	CS	*Hw*	*Pf*	*Pf* density	na	Higher (*P* < 0.001)	Age, sex and splenomegaly status
[33]	Senegal	203 (1–14)	PC	*Hw* + *Al* + *Tt*	*Pf*	*Pf* incidence	na	Higher (AOR: 2.69; 95 % CI: 1.34–5.39)	Age, sex and household
[37]	Nigeria	320 (1–6)	RCT	*Al* + *Hw* + *Tt*	*Pf*	1. *Pf* prevalence 2. Rate of increase in *Plasmodium* density 3. Rate of increase in haemoglobin levels	na	1. Similar (OR: 1.16; 95 % CI: 0.73–1.85) 2. Similar 3. Similar	Age, sex, SES, village and parasitic infection

*Abbreviations*: *Al* *Ascaris lumbricoides*, *CS* Cross-sectional, *Hw* hookworm, *ITN* Insecticide treated nets, *na* not available/not provided/not mentioned/not specified, *OR* odds ratio, *Pf* *Plasmodium falciparum*, *Pv* *Plasmodium vivax*, *RCT* randomized control trial, *RR* Relative risk, *Sh* *Schistosoma haematobium*, *Sm* *Schistosoma manosni*, *STH* soil-transmitted helminths, *Tt* *Trichuris trichiuria*, *SES* socio-economic status, *PC* prospective control

Only seven of the 11 studies were included in the meta-analysis [28, 29, 31–33, 36, 37]. Four studies were cross-sectional and prevalence data reported in these studies were used to estimate the summary odds of *P. falciparum* infection in children infected with STH but uninfected with intestinal helminths. The remaining three studies were longitudinal in nature [31, 33, 37]. Longitudinal studies by Abanyie et al. [31] and Kirwan et al. [37] did not report incidence data, hence prevalence data reported in these studies during the baseline survey [31] and at the end of the follow-up survey [37] were used when estimating the summary odds of *P. falciparum* infection in children infected with STH. In the longitudinal study by Roussilhon et al. [33], the odds ratio for the occurrence of at least one episode of *P. falciparum* malaria during the follow-up was used when estimating the summary odds ratio. As the number of studies included in the meta-analysis were only seven, subgroup analysis was not conducted based on the type of STH species, clinical stage of malaria and study design, while quantifying the summary effect of STH infection on the odds of asymptomatic/uncomplicated *P. falciparum* infection.

Two studies by Kung’u et al. [35] and Kinughi et al. [27] resulted in substantial heterogeneity (*I*^*2*^ = 78.1 %) and were excluded while estimating the summary odds of the effect of STH on *P. falciparum* infection. Some cases of malaria in the study by Kung’u et al. [35] were due to infection with *Plasmodium* species other than *P. falciparum*, and the study by Kinughi et al. [27] did not compare the difference in the outcome between children infected with hookworms and uninfected with intestinal helminths. In addition, two studies by Mboera et al. [30] and Bejon et al. [34] were also excluded from the meta-analysis because they reported data on the incidence [34] and density [30] of *P. falciparum* infection in children infected with STH and uninfected with intestinal helminths. Only one study reported data on the incidence of *P. falciparum* infection [34] and studies reporting *Plasmodium* density did not contain sufficient data to be included in a meta-analysis*.* Thus, summary effects of STH infection on the risk of *P. falciparum* infection or mean *Plasmodium* density were not estimated.

### Association between STH infection and prevalence of *Plasmodium falciparum* malaria

**Fig. 2:**
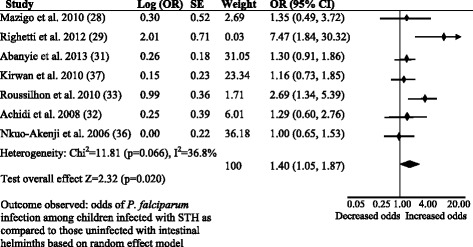
Forest plot. Comparison for the prevalence of asymptomatic/uncomplicated *P. falciparum* infection between children infected with STH and those not infected with intestinal helminths in SSA

Nine studies examined the relationship between *A. lumbricoides*, hookworm or pooled STH species infection with developing asymptomatic/uncomplicated *P. falciparum* infection, and seven of these studies were included in the meta-analysis (Fig. 2). Three cross-sectional studies examined the relationship between hookworm infection and odds of asymptomatic/uncomplicated *P. falciparum* infection [27–29]. Two studies showed similar prevalence of asymptomatic [28] or uncomplicated [27] *P. falciparum* infection in children infected with hookworms compared to those who were not infected with intestinal helminths. However, one study showed an increased prevalence of uncomplicated *P. falciparum* infection in children infected with hookworms compared to those who were uninfected with intestinal helminths [29]. There was only one longitudinal study, which assessed the effect of *A. lumbricoides* co-infection on *P. falciparum* infection. Based on the data available during the baseline survey, the odds of uncomplicated *P. falciparum* infection were similar between children who were infected with *A. lumbricoides* and uninfected with intestinal helminths [31].

Assuming a similarity in the nature of immune responses elicited by the different STH species, some studies evaluated the effect of STH after pooling the different species into one single variable. Two cross-sectional studies in Cameroon reported similar prevalence of asymptomatic [32] or uncomplicated [36] *P. falciparum* infection among children infected with STH and uninfected with intestinal helminths. Likewise, a longitudinal study reported a similar prevalence of uncomplicated *P. falciparum* infection between children who were infected with STH and uninfected with intestinal helminths after 14 months of follow-up in Nigeria [37]. However, a prospective cohort study reported increased odds of uncomplicated *P. falciparum* infection in children infected with any STH species compared to those uninfected with intestinal helminths [33]. The summary OR for asymptomatic/uncomplicated *P. falciparum* infection was 1.4 (95 % CI: 1.05–1.87) among children infected with STH as compared to children uninfected with intestinal helminths. There was moderate degree of heterogeneity between the seven studies (*I*^*2*^: 36.8 %). There was no publication bias among the seven studies included in the meta-analysis. The funnel plot drawn using the odds of asymptomatic/uncomplicated *P. falciparum* infection in children infected with STH as compared to those uninfected with intestinal helminths and the standard error estimates of the odds ratio was symmetric (Additional file 3: Figure S1). Out of the seven studies, only one was outside of the funnel and the Egger’s regression test was not significant (Egger’s test = 2.27, *P* = 0.1).

### Association between STH infection and *Plasmodium falciparum* density

Of the 11 studies included in this review, three reported *Plasmodium* density in children infected with STH and those not infected with intestinal helminths. Two cross-sectional studies showed a higher *Plasmodium* density in children infected with hookworms [30] and heavy intensity of STH infection [36] compared to children without intestinal helminth infection and those with low worm burdens, respectively. *Plasmodium* density also increased at a higher rate among children treated with anthelminthic drug than those treated with placebo every four-months for 14 months [37] but this difference was not statically significant. However, these three studies [30, 36, 37] did not contain sufficient data to estimate the summary effects of STH infection on *Plasmodium* density.

### Association between STH and *Plasmodium falciparum* co-infection and anaemia

**Fig. 3:**
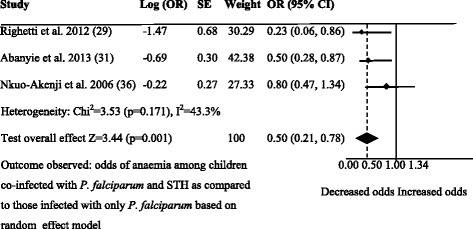
Forest plot. Comparison for the prevalence of anaemia between children co-infected with STH and uncomplicated *P. falciparum* and those infected with *P. falciparum* only

The odds of anaemia were lower in children who were co-infected with *P. falciparum* and hookworm [29] or pooled STH species [31] compared to those who were infected with *P. falciparum* alone. In contrast, two studies reported similar odds of anaemia [36] or mean haemoglobin level [32] between children co-infected with STH and *P. falciparum* and those who were infected with *P. falciparum* alone. A summary OR for anaemia among children co-infected with *P. falciparum* and STH was 0.5 (95 % CI: 0.21–0.78) as compared to those who were infected with *P. falciparum* alone in the three studies [29, 31, 36]. There was however moderate degree of heterogeneity between these three studies, yet the chi-square test was not significant (*I*^2^: 43.3 %, *P* = 0.171) (Fig. 3). After removing one study [36], the heterogeneity decreased significantly (*I*^2^: 10 %, *P* = 0.291). There was no publication bias among the three studies evaluating the odds of anaemia among children co-infected with *P. falciparum* and STH and those who were infected with *P. falciparum*. The funnel plot looks symmetrical and the Egger’s test for the asymmetry was not significant (Egger’s test: −2.67, *P* = 0.395) (Additional file 3: Figure S2).

### Quality of the studies

Information about the quality of the studies included in this review is presented in Table 2. The study design of seven studies was weak (i.e. cross-sectional), however, these studies were strong in controlling for confounders. Nine studies had strong and two studies had moderate quality. All studies used appropriate methodology for data collection. Overall, none of the studies were excluded because of quality issues such as selection bias, weak study design, confounders, lack of blinding, weak data collection methods, withdrawals and dropouts.

**Table 2:** Assessment of the quality of all studies included in the review based on Effective Public Health Practice Project: Quality assessment tool for quantitative studies

Study no.	Author (Year) [Reference]	Selection bias	Study design	Confounders	Blinding	Data collection methods	Withdrawals and drop-outs	Final rating
1	Kinung’hi et al. (2014) [27]	2	3	1	1	1	2	2
2	Mazigo et al. (2010) [28]	2	3	1	1	1	2	2
3	Righetti et al. (2012) [29]	3	3	1	1	1	2	2
4	Mboera et al. (2011) [30]	2	3	1	1	2	2	2
5	Abanyie et al. (2013) [31]	2	1	1	1	1	3	2
6	Achidi et al. (2008) [32]	2	3	2	1	2	2	2
7	Roussilhon et al. (2010) [33]	2	2	1	2	1	1	2
8	Bejon et al. (2008) [34]	2	1	1	2	1	1	2
9	Kung’u et al. (2009) [35]	2	3	2	1	1	2	2
10	Nkuo-Akenji et al. (2006) [36]	2	3	2	1	2	2	2
11	Kirwan et al. (2010) [37]	2	1	1	1	1	3	2

Coding: 1, strong; 2, moderate; 3, weak

## Discussion

Findings from the current systematic review and meta-analysis of 11 studies with 7458 children from seven SSA countries showed that STH infection is associated with an increased prevalence and density of asymptomatic/uncomplicated *P. falciparum* infection but with a decreased occurrence of *P. falciparum* infection-related anaemia. These findings emphasize the complex nature of interaction between STH and *P. falciparum* infection [17, 18, 20]. This could be due to the variability in the mechanisms of interaction between STH and *P. falciparum* within human hosts and the nature of immune response induced during co-infection with both parasite groups [6, 7].

The current results are in agreement with the findings of a meta-analysis by Naing et al. [20], who reported positive association between uncomplicated malaria and STH co-infection among school age children based on studies conducted globally. The findings of positive association between STH and *P. falciparum* infection could be due to the presence of common environmental, socio-economic and behavioral factors that can increase the risk of concurrent infection with both STH and *Plasmodium* spp. [4]. In addition, the increased density of *P. falciparum* infection among children co-infected with STH could be due to downregulation of the immune system [6]. Consequently, the *Plasmodium* parasite could enter into the host and multiply at a faster rate in children co-infected with STH. Another hypothesis is that, increased occurrence of *P. falciparum* in children infected with STH could result from STH-mediated anaemia or lower haemoglobin concentration [4], making individuals more attractive to mosquitoes due to increased lactate and CO_2_ levels [47].

In the present review, the pooled estimates based on three studies [29, 31, 36] showed a lower odds of anaemia in children co-infected with STH and *P. falciparum* than in those infected with *P. falciparum* only. This finding could be due to the immune dependent cause of malarial anaemia, which can be downregulated during STH co-infection. *Plasmodium* infection induces pro-inflammatory cytokines that lower the production of erythropoietin responsible for red blood cell proliferation [48, 49]. On the other hand, STH infection induces the anti-inflammatory cytokine IL-10 that can downregulate the production and activity of pro-inflammatory cytokines [50]. As a result, the risk of anaemia among individuals infected with *P. falciparum* could be reduced when they are co-infected with STH. Moreover, children infected with *P. falciparum* alone could be at increased risk of inflammation, which is associated with anaemia [29, 51]. However, the review by Naing et al. [20] estimated an increased odds of anaemia among non-pregnant adults co-infected with STH and malaria. The disagreement in the findings could be due to the difference among the original studies included in the reviews regarding the type and intensity of STH or *Plasmodium* infection investigated and the age of the study participants [4, 52]. Indeed, a subgroup analysis in the review by Naing et al. [20] showed increased odds of anaemia during hookworm and *Plasmodium* co-infection, but not with *T. trichiura* or *A. lumbricoides* and *Plasmodium* co-infection. Among the three studies included in the current meta-analysis that examined the odds of anaemia among children co-infected with STH and *P. falciparum*, only one study involved co-infection with hookworms.

### Strengths and limitations

This is the first systematic review on this topic and the meta-analysis employed a random effects model to estimate the summary effect. Most effect measures used in estimating the summary effects were adjusted measures, which give strength to the pooled results. In addition, there was no risk of publication bias among the studies included in the meta-analysis. However, this review was not without limitations. Most of the studies included in the review were cross-sectional in nature. Therefore, it was difficult to conclude whether the observed high prevalence and density of *P. falciparum* infections and low prevalence *P. falciparum-*related anaemia were due to STH infection. In addition, there was a moderate level of bias within the studies included in this review. This might have resulted in an overestimation of the evidence of relationship seen between STH and asymptomatic/uncomplicated *P. falciparum* malaria in the current review. Possible diagnostic inaccuracies for STH and malaria in the original studies included in this review may have also affected the present results [53–55]. In addition, the original studies may not have fully controlled the effect of different confounders that could affect the nature of relationship of STH and malaria [52].

## Conclusions

STH infection may increase susceptibility to high-density asymptomatic or uncomplicated *P. falciparum* infection but may protect malaria-related anaemia in children. Future studies should consider the effect of STH infection upon the incidence of uncomplicated or severe *P. falciparum* infection among children in SSA. This review has implications for practice. It suggests that treating children with anthelminthic drugs might decrease the occurrence of asymptomatic/uncomplicated *P. falciparum* infection among children in SSA. But it may lead to an increased prevalence of *P. falciparum* infection-related anaemia among children co-infected with both parasites. The present review did not assess the impact of STH infection upon severe *P. falciparum* malaria. Thus, it is impossible to provide conclusive evidence whether treating of children for STH infection with anthelminthic drugs would be advantageous or detrimental to severe *P. falciparum* malaria. Further investigation on the nature of the interaction between the two parasite groups would be important to provide tangible evidence on the effect of deworming children in SSA on malaria.

## Additional files

Additional file 1: PRISMA checklist. (DOC 64 kb) Additional file 2: Table S1. Search details for the PubMed database. (DOCX 12 kb) Additional file 3
**Figure S1.** Funnel plot. Odds ratio against standard error of odds ratio for seven studies, which compared the prevalence of asymptomatic/uncomplicated *P. falciparum* infection between children who were infected with STH and not infected with intestinal helminth in SSA. **Figure S2.** Funnel plot. Odds ratio against standard error of odds ratio for three studies, which compared the prevalence of anaemia between children who were co-infected with STH and asymptomatic/uncomplicated *P. falciparum* and those infected with only *P. falciparum* in SSA. (ZIP 35 kb)
