# Supplementary material for: Plasmodium falciparum and soil-transmitted helminth co-infections among children in sub-Saharan Africa: a systematic review and meta-analysis
Source: Parasit Vectors. 2016 Jun 15;9:344. doi: 10.1186/s13071-016-1594-2 (PMC4908807; doi:10.1186/s13071-016-1594-2)
Supplement: Additional file 2: Table S1. — Search details for the PubMed database. (DOCX 12 kb) [file 13071_2016_1594_MOESM2_ESM.docx]

Additional file 2: **Table S1**. Search details for the PubMed database

| (("malaria"[MeSH Terms] OR "malaria"[All Fields]) OR ("plasmodium"[MeSH Terms] OR "plasmodium"[All Fields]) OR ("plasmodium falciparum"[MeSH Terms] OR ("plasmodium"[All Fields] AND "falciparum"[All Fields]) OR "plasmodium falciparum"[All Fields]) OR ("plasmodium vivax"[MeSH Terms] OR ("plasmodium"[All Fields] AND "vivax"[All Fields]) OR "plasmodium vivax"[All Fields])) AND (("helminths"[MeSH Terms] OR "helminths"[All Fields] OR "helminth"[All Fields]) OR ("schistosoma"[MeSH Terms] OR "schistosoma"[All Fields]) OR ("schistosoma mansoni"[MeSH Terms] OR ("schistosoma"[All Fields] AND "mansoni"[All Fields]) OR "schistosoma mansoni"[All Fields]) OR ("schistosoma haematobium"[MeSH Terms] OR ("schistosoma"[All Fields] AND "haematobium"[All Fields]) OR "schistosoma haematobium"[All Fields])) AND "humans"[MeSH Terms] |
| --- |
