# Supplementary figures and images for: Plasmodium falciparum and soil-transmitted helminth co-infections among children in sub-Saharan Africa: a systematic review and meta-analysis
Source: Parasit Vectors. 2016 Jun 15;9:344. doi: 10.1186/s13071-016-1594-2 (PMC4908807; doi:10.1186/s13071-016-1594-2)

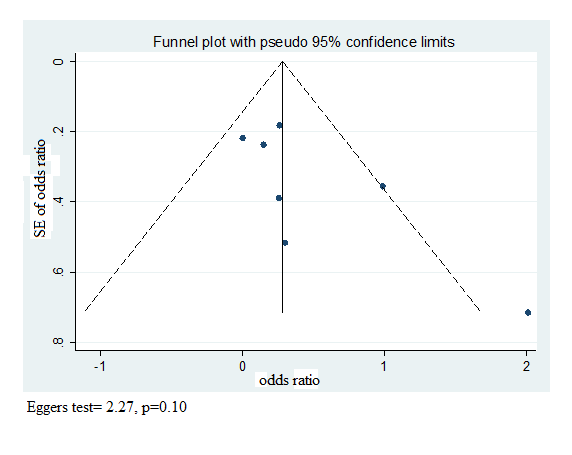

Supplement: Additional file 3 — Figure S1. Funnel plot. Odds ratio against standard error of odds ratio for seven studies, which compared the prevalence of asymptomatic/uncomplicated P. falciparum infection between children who were infected with STH and not infected with intestinal helminth in SSA. Figure S2. Funnel plot. Odds ratio against standard error of odds ratio for three studies, which compared the prevalence of anaemia between children who were co-infected with STH and asymptomatic/uncomplicated P. falciparum and those infected with only P. falciparum in SSA. (ZIP 35 kb) [file 13071_2016_1594_MOESM3_ESM.zip › additional file 3/Additional file 3 Fig. S1.tif]

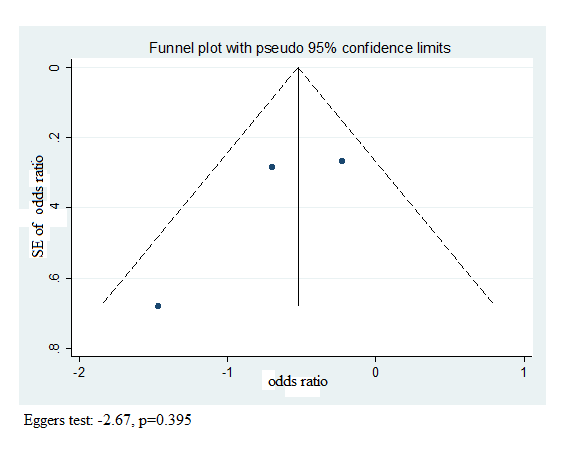

Supplement: Additional file 3 — Figure S1. Funnel plot. Odds ratio against standard error of odds ratio for seven studies, which compared the prevalence of asymptomatic/uncomplicated P. falciparum infection between children who were infected with STH and not infected with intestinal helminth in SSA. Figure S2. Funnel plot. Odds ratio against standard error of odds ratio for three studies, which compared the prevalence of anaemia between children who were co-infected with STH and asymptomatic/uncomplicated P. falciparum and those infected with only P. falciparum in SSA. (ZIP 35 kb) [file 13071_2016_1594_MOESM3_ESM.zip › additional file 3/Additional file 3 Fig. S2.tif]
